# Commentary: A Comment on Qi *et al*.—Authors’ Reply

**DOI:** 10.3390/cancers5010012

**Published:** 2012-12-27

**Authors:** X. Sharon Qi, Qiu Hui Yang, Steve Lee, X. Allen Li, Dian Wang

**Affiliations:** 1 Department of Radiation Oncology, University of California at Los Angeles, 200 ULCA Medical Plaza, Los Angeles, CA 90024, USA; 2 Department of Radiation Oncology, Medical College of Wisconsin, 8701 Watertown Plank Road, Milwaukee, WI 53226, USA

We appreciate the thoughtful comments from Dr. Jack Fowler on our recent manuscript of an estimation of radiobiological parameters for head and neck cancer (HNC) and the clinical implications [1].

Intensity modulated radiation treatment (IMRT) is able to deliver a high conformal dose to the gross tumor and high-risk subclinical disease regions while minimizing irradiation of many critical tissue structures such as parotid glands, spinal cord, mandible, orbits, chiasm and brain in patients with head and neck cancer (HNC). IMRT is as effective as conventional radiotherapy in control of HNC, even though there is no head-to-head randomization comparison between IMRT and conventional radiotherapy to approve equivalence. As a matter of fact, IMRT is now a standard of care in the treatment of HNC. However, it still remains unclear whether or not the prolonged dose-delivering time of IMRT affects control of HNC. This is an important radiobiology question since repair halftime of HNC with different etiologies and viral infections might be different. In addition, multiple types of IMRT planning systems with large variations in dose-delivering time are commercially available.

The primary objective of our study [1] is to evaluate treatment effectiveness of the prolonged dose delivery times associated with different IMRT techniques. Under normal clinical setting, the fraction dose delivery time is normally less than 25 min for head-and-neck (H&N) irradiation. Our analysis therefore mainly focused on the relevant short component of repair halftime. Our study demonstrated that the prolonged fraction delivery times may reduce the radiation treatment effectiveness due to the short repair halftimes for aggressive HNC cell lines (KB and UMSCC-1) selected for this *in vitro* study.

**Figure 1:**
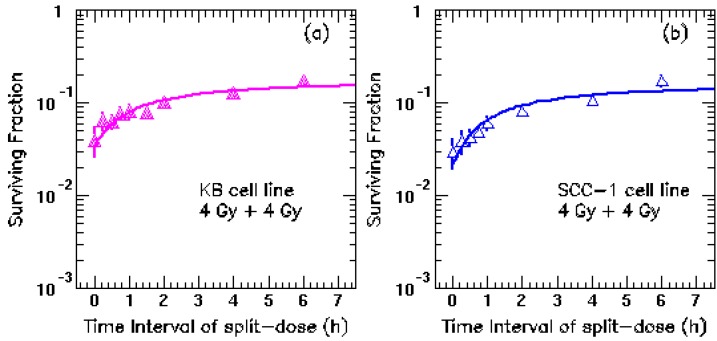
Cell survival fraction as a function of the time interval between split-doses. The split-doses of 4 Gy + 4 Gy were delivered with time intervals of 0, 0.25, 0.5, 0.75, 1.5, 2.0, 4 and 6 hours to the two HNC cell lines. (**a**) KB and (**b**) SCC-1 cell lines. The curves are the fitting results based on LQ model for each cell line separately [1].

As in this study, these two HNC cell lines were irradiated with 4 + 4 Gy fractions, splitted in different intervals from 0 to 6 hours using a 6 MV photon beam generated by a Siemens accelerator. The cell survival fraction (SF) were fitted to the Linear-Quadratic model (LQ) [2,3,4,5] using the least χ^2^ (chi-square) method. The fitting function yielded the repair halftimes are 18 ± 21 and 16 ± 25 minutes for KB and SCC-1 cell line respectively. The goodness of the fitting is measured by the χ^2^ (≈1.0 for both cell lines). Figure 1 shows good agreement of the fitting curves and the measured surviving fraction up to 6 hour interval, with a slightly rising trend in both *in vitro* experiment and our fitted curves. The large error bars are due to the statistical uncertainty calculated by number of survival cells at the each time point.

Although the current fitted curves matches the surviving fractions at different time intervals, a slim rising trend around 4–7 h in Figure 1 might exist, which could be an indicator for slow repair components in cell repair model [2]. However, extensive experiments on the cell survival fractions at longer intervals in a spectrum of HNC cell lines are required to confirm this observation. For example, HNC cell lines with and without HPV infection should also be developed and included in the additional experiments since the biological behaviors and radio-sensitivity of HPV-positive HNC and HPV-negative HNC are significantly different.
